# MicroRNA-Related Polymorphisms in Infectious Diseases—Tiny Changes With a Huge Impact on Viral Infections and Potential Clinical Applications

**DOI:** 10.3389/fimmu.2018.01316

**Published:** 2018-06-14

**Authors:** Joel Henrique Ellwanger, Francis Maria Báo Zambra, Rafael Lima Guimarães, José Artur Bogo Chies

**Affiliations:** ^1^Laboratório de Imunobiologia e Imunogenética, Programa de Pós-Graduação em Genética e Biologia Molecular, Departamento de Genética, Universidade Federal do Rio Grande do Sul (UFRGS), Porto Alegre, Brazil; ^2^Departamento de Genética, Universidade Federal do Pernambuco (UFPE), Recife, Brazil; ^3^Laboratório de Imunopatologia Keizo Asami (LIKA), Universidade Federal de Pernambuco (UFPE), Recife, Brazil

**Keywords:** microRNA, miR, polymorphism, hepatitis B virus, hepatitis C virus, human immunodeficiency virus, Epstein–Barr virus, human papillomavirus

## Abstract

MicroRNAs (miRNAs) are single-stranded sequences of non-coding RNA with approximately 22 nucleotides that act posttranscriptionally on gene expression. miRNAs are important gene regulators in physiological contexts, but they also impact the pathogenesis of various diseases. The role of miRNAs in viral infections has been explored by different authors in both population-based as well as in functional studies. However, the effect of miRNA polymorphisms on the susceptibility to viral infections and on the clinical course of these diseases is still an emerging topic. Thus, this review will compile and organize the findings described in studies that evaluated the effects of genetic variations on miRNA genes and on their binding sites, in the context of human viral diseases. In addition to discussing the basic aspects of miRNAs biology, we will cover the studies that investigated miRNA polymorphisms in infections caused by hepatitis B virus, hepatitis C virus, human immunodeficiency virus, Epstein–Barr virus, and human papillomavirus. Finally, emerging topics concerning the importance of miRNA genetic variants will be presented, focusing on the context of viral infectious diseases.

## Introduction

Viruses are found abundantly in the most diverse environments on earth (1). Some of the animal viruses are responsible for causing human infections. Viral diseases weaken humans individually and have important impacts on the environment, on the social organization and public health systems of populations worldwide. Historically, viruses are responsible for epidemics and outbreaks that impact all nations, being especially a burden in developing countries. Moreover, some viral diseases, such as the acquired immunodeficiency syndrome [AIDS, caused by human immunodeficiency virus (HIV)], affect the entire world, assuming a pandemic characteristic.

Many advances have been made in the combat against viral diseases. Vaccination and antiviral drugs development are examples of medical technologies effectively used against viruses. However, the number of people affected by viral diseases around the world is still alarming. The HIV pandemic alone affects about 37 million people worldwide (2). Our knowledge about the pathogenesis of many viruses is still incipient. Similarly, the natural human defenses against pathogens or the immunogenetic aspects that determine, individually or in terms of a whole population, the degree of susceptibility or resistance to viral infections can still be greatly explored.

Within the context of the host genetics, this review will discuss the impact of microRNA (miRNA)-related polymorphisms on infections caused by hepatitis B virus (HBV), hepatitis C virus (HCV), HIV, Epstein–Barr virus (EBV), and human papillomavirus (HPV). Taking into account the interaction of miRNAs with the epigenetic machinery (3, 4), this review will be relevant to readers interested in epigenetics, genetic polymorphisms, and/or viral diseases.

From this point onward the word “microRNA” will be abbreviated to “miRNA” when we are referring to miRNAs in a general way. However, some clarifications regarding the terminologies used in this review for specific miRNAs are important. miRNAs are named according to the species of which they were derived, indicating it before the prefix “miR,” followed by the identification number of each miRNA (for example, hsa-miR-101 for *Homo sapiens* and mmu-miR-101 for mouse). The prefix “miR” is used to identify mature miRNAs and the prefix “mir” is used to identify precursor hairpins (5, 6). In this review, most cited miRNAs are human-derived mature miRNAs. Thus, the miRNAs quotation was standardized as follows: miR-101, miR-102, miR-103, for example. The few cases of miRNAs encoded by viral genes will be adequately indicated. Besides, in this review, the quotation of the polymorphisms was standardized according to the Single Nucleotide Polymorphism Database (dbSNP) of NCBI (https://www.ncbi.nlm.nih.gov/snp/), based on the reference SNP cluster (rs#) of each polymorphism. Importantly, some authors refer to the forward strand alleles of a given polymorphism while other authors, who studied the same polymorphism, refer to the reverse strand alleles. Although we have standardized the quotations of the SNPs according to the dbSNP, we respect the quotations of the alleles according to the original cited article. Thus, the reader should be aware of this aspect.

## General Aspects of miRNAs

MicroRNAs are small non-coding single-stranded RNA molecules of 19–25 nucleotides in length, well known by its important role in posttranscriptional regulation of gene expression (7). They are present in almost all eukaryotes, including humans, and regulate diverse biological processes in both physiological and pathological conditions (8–10). miRNAs were described to interfere in processes as distinct as cell proliferation and differentiation, apoptosis, or even in viral infections (11, 12). In such infections, the main focus of this review, miRNAs stand up as relevant mediators of the host response, and studies have demonstrated that these molecules can contribute to intracellular defense against the infection, to individual resistance to certain viruses, as well as control the survival, amplification, and modulation of cellular tropism of viruses. On the other hand, also viruses can produce miRNAs. Actually, they use the host cell machinery to generate their own miRNAs (10, 13, 14), which can, for example, to induce viral latency and decrease inflammatory responses, as well as to prevent cell apoptosis, contributing to the oncoviruses-related malignant transformation (15).

**Figure 1:**
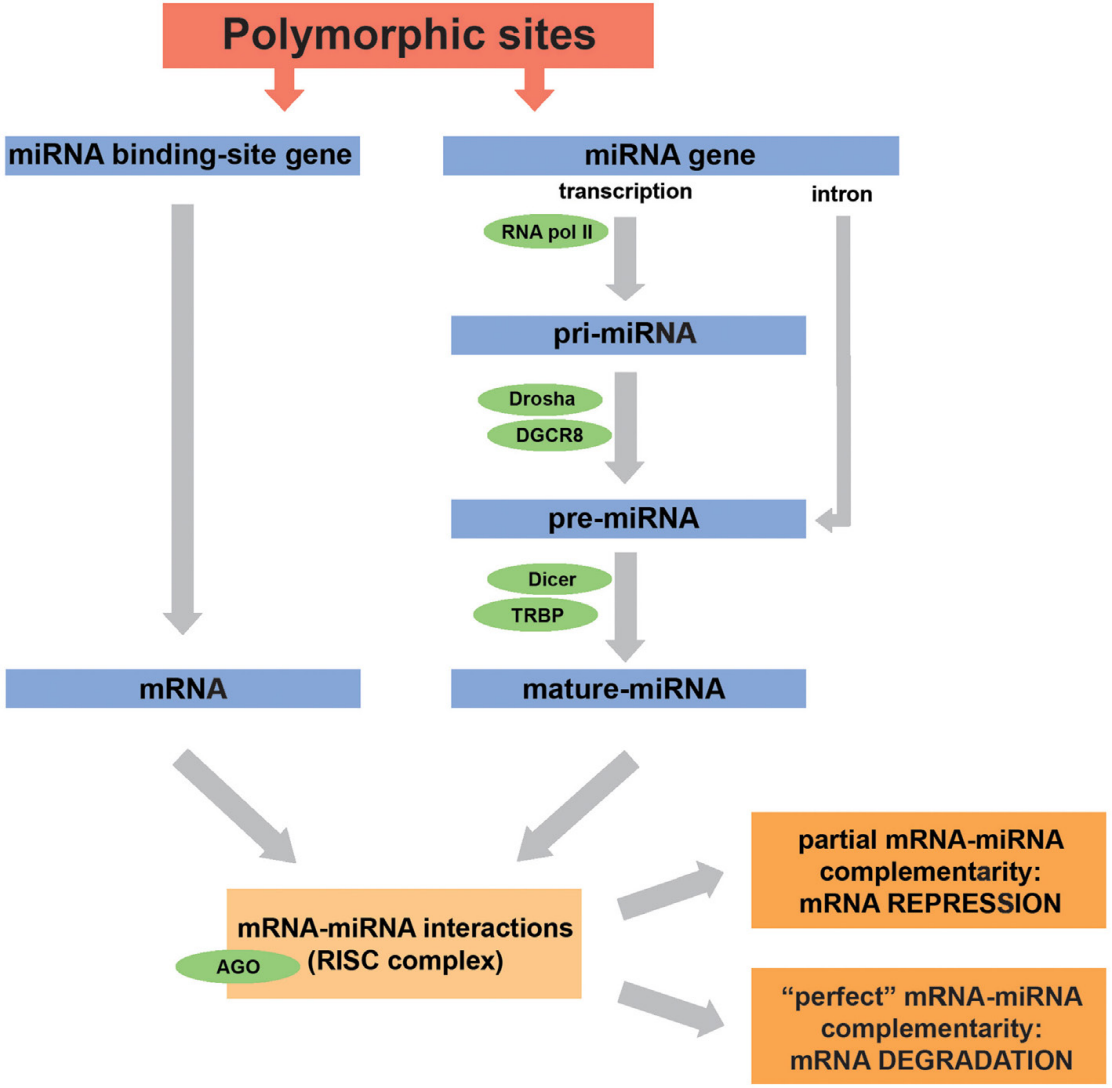
Biogenesis and action of microRNAs (miRNAs) are affected by genetic variations. miRNA biogenesis is mediated by a number of enzymes and co-factors. Some of the most important enzymes for each miRNA processing step are indicated in green. Depending on the degree of miRNA-mRNA complementarity, mRNA expression will be repressed or the molecule will be directed to degradation. Genes encoding miRNAs or miRNA-binding sites can host polymorphisms that modify the action of these molecules. For example, polymorphisms in miRNA genes can downregulate miRNA expression, and polymorphisms in miRNA-binding sites can disrupt the miRNA–mRNA interaction. Furthermore, the effects of genetic variations in miRNA-machinery genes also have an important impact on the biogenesis of miRNAs. This figure was mainly based on the following references: Drury et al. (10), Lorio et al. (21), Ryan et al. (22), Ryan et al. (23), and Rupaimoole and Slack (24).

To understand how polymorphisms can influence the gene expression regulation by miRNAs, and even alter a given biological process, it is important to remember how miRNAs are generated. These molecules can be codified by independent genes or can be inserted in exons or introns from other genes. Briefly, in humans, miRNA biogenesis begins when they are transcribed by the RNA polymerase II as a primary transcript (pri-miRNA), consisting of a molecule encompassing 500–3,000 bases (see Figure 1). In the nucleus, the pri-miRNA is cleaved into pre-miRNA (60–70 nucleotides long) by a complex formed by the Drosha enzyme and its cofactor DGCR8 (DiGeorge syndrome critical region 8 protein) (7). After translocation from nucleus to cytoplasm, a process mediated by the molecule exportin-5 (Exp-5, a nuclear transport factor), pre-miRNAs are cleaved in a mature miRNA (19–25 bases long) by the Dicer/TRBP (trans-activation response RNA-binding protein) complex. Next, the mature single-stranded miRNA and the Argonaut protein (AGO) constitute a multicomponent complex called RNA-induced silencing complex, which allows the binding to complementary sequences in the 3′ untranslated region (3′UTR) of a target mRNA, leading to translational repression or degradation of the mRNA (7, 16–18). The key binding point for miRNA–mRNA interaction is the seed region, located within nucleotides 2–8 from the 5′ end of the mature miRNA sequence (19, 20). In general, a partial complementarity of the mRNA 3′UTR to the miRNA seed sequence leads to translational inhibition, while a perfect complementarity results in mRNA degradation. A slightly distinct process occurs when the miRNA precursor is located in mRNA introns (see Figure 1). In this case, the pre-miRNA will be spliced out and then exported from the nucleus to the cytoplasm, bypassing the Drosha/DGCR8 complex, and then will follow the remaining aforementioned pathway (10).

Polymorphisms in miRNA genes can influence gene transcription, alter the processing of pri- or pre-miRNA, and affect miRNA–mRNA interactions. Moreover, such miRNA–mRNA interactions can also be either facilitated or hindered by polymorphisms located in the 3′UTR of the target mRNAs, by the generation or loss of miRNA-binding sites, for example (25). The effect of gene regulation by miRNAs is quite complex, since a certain miRNA can target several mRNAs, and conversely, a single mRNA can bind to distinct miRNAs, being the final effect determined by the joint action of, potentially, several miRNAs (26). Despite best known by their capacity to impair translational processes, decreasing the rates of protein expression, miRNAs, in some cases, can bind to 5′ untranslated regions, to exons, or even to DNA elements, leading to increased transcription or translation (27–29).

An important emerging research topic concerns the effects of polymorphisms in miRNAs and its target-sites in viral-associated diseases. Recent case–control and functional studies have pointed out to a role of such polymorphisms in susceptibility to viral infection, as well as in chronicity of the disease versus viral clearance, and even in viral-associated cancer development (30–33). Given the increasing interest in such processes and phenomena, in addition to the potential clinical use of miRNAs as molecular biomarkers and therapeutic targets, we will present a review of the existing literature on these topics.

## Viral Infections and Polymorphic Variants That Affect miRNAs

### HBV Infection, HBV-Associated Diseases, and miRNAs

It was estimated that around 30% of the world’s population is a HBV carrier or has been infected with the virus in the past (34). HBV infection is classically responsible by triggering several types of liver damage, including cirrhosis and hepatocellular carcinoma (HCC) (34). Africa and Asia concentrate the largest number of countries with high prevalence of chronic HBV infection (34), and particularly in China, HBV infection is an endemic problem (35). Although several advances in the fight against HBV have been made, a large part of the Chinese population still suffers from HBV-associated diseases (36). Therefore, it was not a surprise, when the literature regarding the influence of miRNA-related polymorphisms on HBV-associated diseases was reviewed, that a large number of studies were performed with individuals from China.

In order to give a comprehensive idea of the studies approaching miRNA polymorphisms, minimizing the potential problems of comparing ethnically distinct populations, we will initially focus on studies performed in China; all other studies being gathered in the next section. Nevertheless, even considering only those studies, and centering in human populations with a relatively homogeneous ethnic origin, conflicting data arouses. In a research performed by Xu et al. (37), the GG genotype of miR-146a G/C SNP (rs2910164) was associated with increased risk of HCC in males. Their study compared 479 HCC patients with 504 controls. Of note, 88.9% of the HCC patients were infected with HBV. Moreover, through *in vitro* assays, the same authors described how miR-146a rs2910164 would be linked to HCC. Briefly, the G allele increases miR-146a maturation, potentially contributing to HCC-related cell proliferation (37). A number of authors reported no impact of miR-146a rs2910164 on HBV-associated HCC (38–43). On the other hand, according to Cong et al. (44), the GG genotype and G allele of miR-146a rs2910164 increase the risk of HCC among HBV-infected individuals. In a recent meta-analysis including eleven studies performed in Chinese populations, the miR-146a rs2910164 was linked to an increased risk of HBV-associated HCC (45). Besides HCC development, other HBV-associated diseases are potentially influenced by this polymorphism. For example, Jiang et al. (46) investigated the miR-146a rs2910164 in patients with acute-on-chronic hepatitis B liver failure and in individuals with chronic HBV infection. Individuals carrying the GG genotype had reduced susceptibility to the disease, lower levels of TNF-α, and higher survival rate (46).

Xiang et al. (38) genotyped the miR-499a C/T SNP (rs3746444) in chronic HBV-infected individuals, HCC patients (HBV-infected and non-infected), and controls. They identified the CC genotype as a risk factor for the development of HBV-associated HCC (38). Posteriorly, and in conflict with the data from the previously cited work, a small case–control study found, in a dominant model, that AG + GG genotypes of miR-499a rs3746444 were associated with a reduced risk of HCC when HBV-infected patients were analyzed (47). In addition, another study with a small sample size reported an increased risk of HBV-associated HCC linked to the A allele of miR-499a rs3746444 (42). Ma et al. (48) investigated the miR-499 rs3746444 and the miR-423 A/C/T (rs6505162) SNP in 984 HCC patients and compared the genotype frequencies with a similar number of controls. Of note, among the HCC group, 760 individuals were infected with HBV. MiR-423 rs6505162 had no effect on HCC risk, independently of the HBV infection status. However, miR-499a TC + CC (in a dominant model) increased the risk of HBV-associated HCC, when compared to the TT genotype (48). Finally, a meta-analysis including case–control studies reinforced the involvement of miR-499a rs3746444 in the susceptibility to HCC among HBV-infected individuals (49). Nevertheless, it is important to consider that several authors did not found a statistically significant link between miR-499a rs3746444 and HBV-associated HCC (39, 41, 43, 50, 51). This fact evidences the need for new investigations aiming to establish with more robustness the impact of this SNP on HBV-associated HCC and reinforces the fact that, in multifactorial diseases, multiple variants of susceptibility can be identified, each of them with a small contribution.

In a study evaluating the miR-196a2 C/T SNP (rs11614913), Qi et al. (52) genotyped 199 chronic HBV-infected individuals without HCC, 361 chronic HBV-infected individuals with HCC, and 391 healthy controls. An increased risk of HBV-associated HCC was found in males carrying the C allele and the CC genotype. Regarding HCC progression, no statistically significant influence of miR-196a2 rs11614913 on tumor number, size, growth phase, stage, and lymph node metastasis was found. However, when stratified by sex, in male patients with lymphatic metastasis, a higher frequency of the T allele was observed (52).

The potential role of miR-196a2 rs11614913 on the risk of HBV-associated HCC was investigated by a number of authors. In a study performed by Hao et al. (39), CT and TT genotypes of miR-196a2 rs11614913 were considered risk factors for HCC development in HBV-infected individuals. In addition, the influence of miR-196a2 rs11614913 on HCC risk was investigated by Li et al. (43) in a small case–control sample (266 individuals in each group). 110 individuals from the HCC group and 32 individuals from the control group were HBV infected. Looking at these individuals, it cames out that CT + TT genotypes increase the risk of HCC development (43), a finding in line with the study performed by Hao et al. (39). However, conflicting results were also published. Kou et al. (41) evaluated this same miRNA variant site in 532 controls and 271 HCC patients. Approximately, 58% of the patients were HBV infected, and CT and TT genotypes presented a reduced risk of HCC (41). Furthermore, Zhang et al. (40) evaluated the miR-196a2 rs11614913 in a relatively large sample of the Chinese population. Their study included a control group (~1,000 individuals) and a group of HCC patients (~1,000, including 771 HBV-associated HCC patients). In brief, CT + TT genotypes and the T allele were linked to a lower chance of HBV-associated HCC development (40). Supporting this result, a small case–control study described CT and TT genotypes as well as the T allele of miR-196a2 rs11614913 as markers of reduced risk of HBV-associated HCC (53). Recently, the miR-196a2 rs11614913 was associated with a decreased risk of HBV-associated HCC in a meta-analysis including eleven studies carried out with Chinese populations (45). A previous meta-analysis (51), approaching a total of 2,693 HCC cases and 3,594 controls, had already associated the T allele and the TT genotype with reduced risk of HCC. Interestingly, this finding had been observed only considering the total pool of individuals, but not when stratifying the populations according to ethnicity (51). Actually, there are studies in Chinese populations where no statistically significant association between the miR-196a2 rs11614913 and risk of HBV-associated HCC were observed [see Yan et al. (54), for example], although these results seem to have been “diluted” with the inclusion of new studies in the more recent meta-analysis.

Another important point to be discussed refers to the interactions between viruses and host genetic factors. To highlight this point let’s take the study from Han et al. (30), which, using quantitative PCR, explored the interaction of miR-196a2 rs11614913 and miR-34b/c T/C SNP (rs4938723) with HBV mutations in a sample of 3,325 individuals (1,021 of them with HBV-associated HCC). Among several results, the most interesting finding was that the effects caused by miRNA SNPs on HBV-associated HCC susceptibility can be strongly influenced by HBV mutations (30). Thus, host genetic polymorphisms may be relevant in the presence of an infection associated to a specific HBV genotype, but less important in the presence of HBVs showing different genetic features. In this sense, conflicting findings in studies evaluating the same particular host polymorphism in the context of HBV-associated diseases may be due not only to differences in the ethnic background of the studied population, but can also result from the HBV genetic variants circulating in this given population.

Some SNPs were studied in a particular context or population and few (or no) further studies were performed to confirm or refute these initial results. Wang et al. (55) investigated the influence of miR-608 C/G SNP (rs4919510) and miR-149 C/T SNP (rs2292832) on the risk of HCC development. No link between miR-608 rs4919510 and HBV-associated HCC was reported. On the other hand, in men, the TT genotype of miR-149 rs2292832 was associated with an increased chance of HBV-associated HCC development when compared to the wild-type genotype (55). Differently, but also evaluating the miR-149 rs2292832, Liu et al. (56) found that the TC + CC genotypes, when compared with TT genotype, increased the risk of HCC in HBV-infected individuals. No link between miR-149 rs2292832 and HBV-associated HCC was reported in other studies (43, 50).

Wang et al. (57) investigated the miR-646 G/T SNP (rs6513497) in HCC patients and controls. Among the 771 HCC patients enrolled in the study, 81.1% were infected with HBV. Among males, the GT genotype and G allele were considered as protective factors against HBV-associated HCC (57). In this same direction, miR-378a C/T SNP (rs1076064) was also described as a protective factor of HBV-associated HCC. Specifically, AG + GG genotypes were associated with a decreased risk of HCC and higher HCC survival rate (58). Of note, these results were attributed, at least partially, to the effects that miR-378a rs1076064 exerts on miR-378 transcription (58). Although the results regarding miR-646 rs6513497 and miR-378a rs1076064 are quite interesting, the lack of confirmatory cohorts hinders further conclusions.

As the miR-122 expression is reduced in tissue samples of HBV-associated HCC (59), Liu et al. (60) evaluated the role of miR-122 A/C SNP (rs4309483) and miR-122 C/T SNP (rs4503880) on the risk of HCC. Their study included 1,300 HBV-infected patients with HCC, 1,344 HBV-infected patients without HCC, and 1,344 patients showing HBV clearance. The expression of pri-miR-122 and mature-miR-122 was measured in 29 HCC patients, comparing the levels in tumoral liver tissue and in adjacent tumor-free regions. In short, based on genotypes and gene expression, the authors concluded that miR-122 rs4309483 increases the risk of HBV-associated HCC (60). On the other hand, the same authors reported this SNP also acts as a protective factor against chronic HBV infection (60). This can be interpreted as follows: miR-122 rs4309483 hampers chronic HBV infection, but if the infection is established, this same SNP facilitates carcinogenesis.

Liu et al. (61) focused their investigation on the MCM7 C/T SNP (rs999885). Importantly, *MCM7* gene is the location of the miR-106b, miR-93 and miR-25 cluster (miR-106b-25) (61). They evaluated the influence of MCM7 rs999885 on the clinical outcome of HBV infection. In addition, the expression of miR-106b-25 was measured both in the HCC tissue and in adjacent tumor-free liver regions of 25 HBV-infected patients. AG/GG genotypes were associated to a higher expression of miR-106b-25 and a higher risk of HBV-associated HCC. Interestingly, these same genotypes were linked to lower risk of chronic HBV infection (61). The impact of MCM7 rs999885 of miR-106b-25 cluster on the outcome of HBV-associated HCC was also studied by Qi et al. (62). In summary, these authors observed that the AG/GG genotypes and G allele of MCM7 rs999885 were linked to a better HCC prognostic (62).

Zhou et al. (63) studied the GAGA ins/del polymorphism (rs17875871) of the 3′UTR of *IFNAR1* gene in a sample of HCC individuals and controls (*n* = 420 in each group). This polymorphism potentially affects the miR-1231-binding site. The deletion allele was associated with an increased risk of HCC, especially in the presence of HBV (63). The influence of polymorphisms in genes that affect the biogenesis/binding of miRNAs was also subject of study of Liu et al. (64). Specifically, polymorphisms in *DICER1, RAN, PIWIL1* genes (C/T rs1057035, A/C/G rs3803012, and C/T rs10773771, respectively) were genotyped in HBV-infected individuals with different clinical outcomes. Of note, DICER1 rs1057035 affects the miR-574-3p binding, RAN rs3803012 impacts the miR-199a-3p binding, and PIWIL1 rs10773771 influences the miR-1264 binding. The impact of the SNPs on the binding of these specific miRNAs was also tested *in vitro*. In brief, the authors found evidence that CT/CC genotypes of both DICER1 rs1057035 and PIWIL1 rs10773771 decreased the risk of HBV-associated HCC. Differently, RAN rs3803012 AG/GG genotypes were a risk factor of HBV persistent infection (64).

Xiong et al. (65) studied the KRAS G/T SNP (rs712), a genetic variation with implications to the binding of miR-let-7 and miR-181. According to these authors, the TT genotype increases the risk of HBV-associated HCC. This influence occurs possibly by a modified expression of *KRAS* due to the rs712-induced changes in the miR-let-7-binding site (65). Li et al. (66) explored the effect of five SNPs in miRNA-binding sites (located at *RAD52* gene) on the risk of HBV-associated HCC. The SNPs analyzed were: RAD52 A/G (rs1051669), RAD52 A/T (rs10774474), RAD52 A/T (rs11571378), RAD52 G/T (rs7963551), and RAD52 C/T (rs6489769). The C allele of RAD52 rs7963551 reduced the risk of HCC development. Of note, this SNP may affect the binding of miR-let-7. The authors also showed that CC or AC genotypes of RAD52 rs7963551 were associated with an increased RAD52 expression. Due to the role of RAD52 in DNA repair, changes in its expression or regulation caused by polymorphisms affecting the miRNA-binding sites may have a significant impact on the risk of HBV-associated HCC (66).

Zhang et al. (67) investigated the impact of PD1 A/G SNP (rs10204525) on the binding of miRNAs in the context of susceptibility to HBV-associated diseases. In summary, their results suggest that the *PD-1* regulation by miR-4717 is modified in response to PD1 rs10204525 genotypes. For example, *in vitro* experiments showed miR-4717 decreased *PD-1* expression in lymphocytes isolated from patients showing chronic HBV infection and GG genotype of PD1 rs10204525. In addition, this phenomenon was found in association with increased levels of TNF-α and IFN-γ. Together, these events may have an important impact on the HBV infection clinical course (67).

The influence of variations in genes of the miRNA machinery on chronic HBV infection was investigated by Shang et al. (68). Such study specifically addressed the following SNPs: DGCR8 A/G (rs3757), AGO1 A/G (rs636832), and GEMIN4 C/T (rs7813). The A allele of AGO1 rs636832 decreased the risk of chronic HBV infection. Moreover, compared to the AA genotype, AG + GG increased the risk of chronic HBV infection, suggesting the AA genotype as a protective factor to the disease. No statistically significant associations were reported in relation to the other analyzed SNPs (68).

In summary, it is evident that polymorphisms can interfere with the maturation and/or in the action of miRNAs, modifying the risk of HBV-associated diseases. Therefore, it is important not only focus on genes that actually encode miRNAs or their binding sites, but also on those miRNA maturation/action modifier genes.

The interaction of a miR-122-binding site TTCA ins/del polymorphism (rs3783553, located at the *IL-1A* gene) and HBV mutations was investigated, in the context of HBV-associated HCC, by Du et al. (69). Interestingly, the TTCA insertion allele was linked to an increased frequency of the HBV C7A mutation. In general, rs3783553 did not modify the risk of HBV-associated HCC, but its interaction with HBV preS deletion reduced the risk of HCC development (69). According to the authors, host genetic polymorphisms influence the risk of HCC more subtly than the influence exerted by the genetic features of HBV. However, there is a strong interaction between viral and host genetic factors defining the course of HBV infection (69). Similar to Du et al. (69), Han et al. (70) evaluated the risk of HBV-associated HCC diseases taking into consideration virus–host interactions, meaning miR-218-2 A/G SNP (rs11134527) and HBV mutations. Briefly, miR-218-2 rs11134527 modified the risk of HCC, cirrhosis development, inflammation, and HBV clearance. Moreover, and again similar to the findings of Du et al. (69), the host genetic variation was associated with HBV preS deletion in men (70). However, in the study performed by Han et al. (70), the interaction of miR-218-2 rs11134527 with HBV preS deletion was linked to an increased risk of HCC. Finally, the T1674C/G HBV mutation reduced the increased risk of HCC linked to miR-218-2 rs11134527 (70). The results of these two studies exemplify the complex relationships between viral and host genetic factors. Besides, it is necessary to study the influence of gene–gene and gene–environment interactions to better understand the effect of miRNA SNPs on HBV-associated HCC (51).

Considering all articles mentioned above, we note that only a few miRNA SNPs have been studied in depth. This is the case of miR-146a G/C SNP (rs2910164) and miR-196a2 C/T SNP (rs11614913). The influence of these genetic variants on HBV-associated diseases is relatively well studied, at least in Chinese populations. However, even in these cases, conflicting results arouse. In order to synthesize the information described in this topic, the main interactions between miRNA SNPs and HVB-associated diseases were compiled in Table 1. In addition to data from studies performed with populations from China, Table 1 also shows information obtained from studies performed in other populations. These studies will be discussed in the next topic.

**Table 1:** Main microRNA (miRNA)-related polymorphisms showing statistically significant influence on hepatitis B virus (HBV) infection and HBV-related diseases.

miRNA or miRNA-binding site^a^	Polymorphism^b^	Influence on	Population	Reference
miR-146a	C/G rs2910164	Susceptibility to HBV infection	Chinese	Cong et al. (44)
		HBV-associated hepatocellular carcinoma (HCC)	Chinese	Zhou et al. (29); Cong et al. (44)
			Meta-analysis	Tian et al. (45)
		Acute-on-chronic hepatitis B liver failure	Chinese	Jiang et al. (46)
		Susceptibility to HBV infection; HBV clearance	Saudi Arabian	Al-Qahtani et al. (33)

miR-149	C/T rs2292832	HBV-associated HCC	Chinese	Wang et al. (55); Liu et al. (56)
			Korean	Kim et al. (71)
		Susceptibility to HBV infection; HBV clearance; HBV persistence; HBV-associated cirrhosis/HCC	Saudi Arabian	Al-Qahtani et al. (33)

miR-499	C/T rs3746444	HBV-associated HCC	Korean	Kim et al. (71)
			Chinese	Xiang et al. (38); Li et al. (42); Zou and Zhao (47); Ma et al. (48)
			Meta-analysis	Yu et al. (49)

miR-196a2	C/T rs11614913	HBV-associated HCC	Chinese	Hao et al. (39); Zhang et al. (40); Kou et al. (41); Li et al. (43); Qi et al. (52); Zhou et al. (53)
			Meta-analysis	Tian et al. (45); Zhu et al. (51); Xu et al. (72)
			Turkish	Akkiz et al. (73)
		Gene-gene interaction; HCC-related HBV mutations	Chinese	Han et al. (30)
		Susceptibility to HBV infection; HBV clearance; HBV-associated cirrhosis/HCC	Saudi Arabian	Al-Qahtani et al. (33)

miR-196a2	A/C rs12304647	HBV-associated HCC	Korean	Kim et al. (74)
		HBV-associated cirrhosis/HCC	Malaysian	Riazalhosseini et al. (75)

miR-34b/c	C/T rs4938723	Gene-gene interaction; HCC-related HBV mutations	Chinese	Han et al. (30)

miR-423	A/C/T rs6505162	HBV clearance; HBV-associated cirrhosis/HCC	Saudi Arabian	Al-Qahtani et al. (33)

miR-26a1	C/T rs7372209	HBV-associated cirrhosis/HCC	Saudi Arabian	Al-Qahtani et al. (33)

miR-608	C/G rs4919510	HBV-associated cirrhosis/HCC	Saudi Arabian	Al-Qahtani et al. (33)

miR-492	C/G rs2289030	HBV clearance	Saudi Arabian	Al-Qahtani et al. (33)

miR-30a	A/G rs1358379	Susceptibility to HBV infection; HBV clearance; HBV persistence; HBV-associated cirrhosis/HCC	Saudi Arabian	Al-Qahtani et al. (33)

miR-122	A/C rs4309483	Chronic HBV infection; HBV-associated HCC	Chinese	Liu et al. (60)

miR-122-binding site	ins/del rs3783553	HCC-related HBV mutations	Chinese	Du et al. (69)

miR-371-372-373 cluster	A/C rs3859501	HBV-associated HCC	Korean	Kwak et al. (76)

miR-106b-25 cluster	C/T rs999885	HBV-associated HCC	Chinese	Liu et al. (61); Qi et al. (62)
		Chronic HBV infection	Chinese	Liu et al. (61)

miR-101-1	C/G/T rs7536540	HBV-associated cirrhosis/HCC	Korean	Bae et al. (77)

miR-101-2	C/T rs12375841	HBV clearance	Korean	Bae et al. (77)

miR-1231-binding site	ins/del rs17875871	HBV-associated HCC	Chinese	Zhou et al. (63)

miR-219-1	A/G rs107822	HBV clearance	Korean	Cheong et al. (78)

miR-219-1	C/T rs421446	HBV clearance	Korean	Cheong et al. (78)

miR-219-1	C/T rs213210	HBV clearance	Korean	Cheong et al. (78)

miR-574-3p-binding site	C/T rs1057035	HBV-associated HCC	Chinese	Liu et al. (64)

miR-1264-binding site	C/T rs10773771	HBV-associated HCC	Chinese	Liu et al. (64)

miR-199a-3p-binding site	A/C/G rs3803012	HBV-associated HCC; HBV persistance	Chinese	Liu et al. (64)

miR-378	C/T rs1076064	HBV-associated HCC	Chinese	An et al. (58)

miR-604	C/T rs2368392	HBV-associated HCC; HBV persistance	Korean	Cheong et al. (79)

miR-218	A/G rs11134527	Gene-gene interaction; HCC-related HBV mutations; HBV-associated cirrhosis/HCC; HBV clearance	Chinese	Han et al. (70)

miR-646	G/T rs6513497	HBV-associated HCC	Chinese	Wang et al. (57)

miR-let-7-binding site	G/T rs7963551	HBV-associated HCC	Chinese	Li et al. (66)

miR-let-7-binding site	G/T rs712	HBV-associated HCC	Chinese	Xiong et al. (65)

miR-4717-binding site	A/G rs10204525	Chronic HBV infection	Chinese	Zhang et al. (67)

miR-323b	A/C/T rs56103835	HBV persistence	Korean	Yu et al. (80)

*^a^Seed or regulatory region*.

*^b^Polymorphism quotations were standardized according to the Single Nucleotide Polymorphism Database (dbSNP) of NCBI (https://www.ncbi.nlm.nih.gov/snp/), based on the reference SNP cluster (rs#) of each polymorphism*.

### HBV: More Studies With Diverse Human Populations

The potential role of miR-196a2 C/T SNP (rs11614913) on HBV-associated diseases was addressed by different authors in distinct human populations comprising ethnic backgrounds other than Chinese (focus of the previous topic). Data from a small case–control study performed by Akkiz et al. (73) in a Turkish population, pointed the C allele and the CC genotype as potential markers to identify individuals at high risk for developing HBV-associated HCC who could benefit from more frequent HCC preventive examinations. However, conflicting results regarding the effects of such variant were published later. Kim et al. (74) studied in a Korean population the impact of miR-196a2 rs11614913 and miR-196a2 A/C SNP (rs12304647) on the clinical outcome of HBV infection. In addition to 404 patients with HBV spontaneous recovery, the study included 313 HBV-infected patients with chronic hepatitis, 305 HBV-infected patients with liver cirrhosis, and 417 HBV-patients with HCC, in a total of 1,035 HBV-infected individuals. Briefly, among HBV-infected patients with chronic hepatitis or cirrhosis, the CC genotype of miR-196a2 rs12304647 was linked to a reduced risk of HCC, although no statistically significant influence of the miR-196a2 rs11614913 on HCC development was observed (74).

In a case–control study, Riazalhosseini et al. (75) genotyped three polymorphisms in three Malaysian ethnical groups (Malays, Chinese, and Indians): miR-196a2 C/T SNP (rs11614913), miR-196a2 A/C SNP (rs12304647), and miR-146a C/G SNP (rs2910164). The authors evaluated the influence of these SNPs on the development of HBV-associated cirrhosis and HCC, comparing 103 chronic HBV-infected patients with liver cirrhosis or with cirrhosis and HCC to 423 chronic HBV-infected patients without such conditions. No statistically significant influence of miR-196a2 rs11614913 and miR-146a rs2910164 on the HBV-associated diseases was observed. However, when compared to CC genotype, AA + AC genotype of miR-196a2 rs12304647 was linked to a reduced risk of cirrhosis/HCC (75).

Kim et al. (71) investigated in a case–control study with a Korean population the role of miR-196a2 C/T SNP (rs11614913), miR-149 C/T SNP (rs2292832), miR-146a C/G SNP (rs2910164), and miR-499a C/T SNP (rs3746444) on the risk of HCC development. Among 159 HCC patients, 127 were HBV infected. In relation to miR-149 rs2292832, CT genotype and CT + CC in a dominant model reduced the risk of HCC in HBV-infected and non-infected individuals. Considering miR-499a rs3746444, an AG + GG model also reduced the risk of HBV-associated HCC. No influence on HBV-associated HCC was observed for miR-146a rs2910164 and miR-196a2 rs11614913 in this study (71), although a meta-analysis (72) suggested that the miR-146a rs2910164 C allele decreases the risk of HCC in populations with an Asian ethnic background and also in Caucasians. No effect of miR-499a rs3746444 was observed in this same meta-analysis (72).

The influence of miR-149 C/T SNP (rs2292832) and miR-101-1 C/G/T SNP (rs7536540) on the risk of HCC in Thai population was evaluated by Pratedrat et al. (81), in a study including 95 healthy controls, 90 chronic HBV-infected individuals, and 104 HCC patients. However, no statistically significant association was found (81). In addition to miR-101-1 rs7536540, the influence of the following variants on clinical outcome of HBV infection was investigated in Korean individuals (77): miR-101-2 C/T SNP (rs17803780), miR-101-2 C/T SNP (rs12375841), and miR-338 C/T SNP (rs62073058). In brief, miR-101-1 rs7536540 had an impact on the risk of liver cirrhosis and HCC, and miR-101-2 rs12375841 and the haplotype ht2 (T-C) of miR-101-2 influenced the HBV clearance (77).

The role of three variants of the miRs-371-372-373 cluster (C/T SNP rs28461391, A/C rs3859501, and C/T rs12983273) on the risk of HCC and HBV clearance was investigated by Kwak et al. (76) in a sample of 1,439 Korean individuals. The miRs-371-373 rs3859501 and the ht2 (C-A-C) haplotype were linked to a reduced risk of HBV-associated HCC. However, no statistically significant influence of those SNPs was observed concerning HBV clearance (76). Another study from Korea evaluated the impact of three distinct variants of miR-219a1 (C/T rs421446, A/G rs107822, and C/T rs213210) on HBV clinical outcome (78). In brief, all SNPs evaluated and the ht1 (C-A-C) and ht2 (T-G-T) haplotypes showed some influence on HBV clearance. Conversely, no statistically significant influence of those SNPs on HBV-associated HCC was reported. These results indicate that miR-219a1 has an important influence specifically on HBV clearance. However, the mechanisms by which miR-219a1 acts on HBV infection and how its SNPs can affect those mechanisms are still unclear and may be subject to functional studies (78). Posteriorly, the T allele of miR-604 C/T SNP (rs2368392) was linked to HBV chronic infection in Korean patients (79), although, unexpectedly, in patients chronically infected with HBV this allele reduced the risk of HCC occurrence (79). In other words, this SNP seems to play a role in the maintenance of the infection, but it does not necessarily contribute to the mechanisms of hepatocarcinogenesis.

Still considering Korean patients, Yu et al. (80) evaluated the miR-323b A/C/T SNP (rs56103835) on HBV replication and clinical course of infection. In that study, miR-323b rs56103835 was associated with persistent infection and was hypothesized as a factor which facilitates chronic HBV infection. In line with this interpretation, this SNP promoted HBV replication *in vitro* (80). In association, these findings support an important role for miR-323b rs56103835 in HBV chronic infection, once miR-323b can be considered an HBV suppressor (80). Of note, some points in the study of Yu et al. (80) (statistical analysis and interpretation of results) were target of criticism (82) which should be taken into account when interpreting the results mentioned above.

Recently, Al-Qahtani et al. (33) investigated the role of a number of miRNA SNPs on HBV-associated liver diseases in Saudi Arabia, including 1,352 HBV-infected patients and 600 healthy HBV uninfected controls. The genotyped variants were: miR-499a C/T SNP (rs3746444), miR-423 A/C/T SNP (rs6505162), miR-26a1 SNP C/T (rs7372209), miR-608 C/G SNP (rs4919510), miR-604 C/T SNP (rs2368392), miR-492 C/G SNP (rs2289030), miR-149 C/T SNP (rs2292832), miR-146a C/G SNP (rs2910164), miR-196a2 C/T SNP (rs11614913), and miR-30a A/G SNP (rs1358379). Briefly, the authors evidenced that the polymorphisms of miR-149 rs2292832, miR-146a rs2910164, miR-196a2 rs11614913, and miR-30a rs1358379 were significantly more frequent in patients than in the control group (33). As a remark, Cong et al. (44) have already described that miRNA-146a rs2910164 may be involved in immune regulation during HBV infection in a Chinese population. Moreover, in this same study miR-30a rs1358379, miR-149 rs2292832, miR-146a rs2910164, miR-423 rs6505162, miR-492 rs2289030, and miR-196a2 rs11614913 were associated to HBV clearance (33). HBV persistence was impacted by miR-149 rs2292832 and miR-30a rs1358379. Finally, miR-196a2 rs11614913, miR-30a rs1358379, miR-26a1 rs7372209, miR-608 rs4919510, miR-149 rs2292832, and miR-423 rs6505162 impact the development of HBV-associated cirrhosis, or HBV-associated HCC. No statistically significant associations were reported concerning miR-604 rs2368392 or miR-499a rs3746444 on HBV-associated diseases (33). Of particular interest, the finding regarding miR-499a rs3746444 corroborates the previously mentioned study of Xu et al. (72).

Behelgardi et al. (83) studied the influence of IL-16 T/C (rs1131445), an SNP located in a miRNA-binding site in 3′UTR of the *IL-16* gene, and the risk of HBV chronic infection in an Iranian population. After adjustment for covariates, including age and gender, the TC genotype was associated with an increased risk of HBV chronic infection. IL-16 is a pro-inflammatory cytokine that activates T cells, monocytes, dendritic cells, and macrophages, as well as stimulates other pro-inflammatory cytokines, such as IL-1β, IL-6, and IL-15. Thus, polymorphisms that modulate the production of IL-16 could be important regulators of susceptibility to viral infections (83).

### Hepatitis C Virus

The susceptibility to HCV infection as well as the progression of HCV-related diseases result from the interaction of host and viral genetic characteristics, and are mediated by environmental and different physio-metabolic factors (84). Focusing on the host genetics, the importance of miR-146a G/C SNP (rs2910164) (44, 53) and miR-196a2 C/T SNP (rs11614913) (39, 41, 53, 54) on HCV-associated disease was investigated in Chinese individuals. Despite these efforts, no statistically significant association was found between the SNPs and HCV-associated diseases (39, 41, 44, 53, 54). Furthermore, no statistically significant association between miR-196a2 rs11614913 and HCV-related HCC was reported in an investigation encompassing the Turkish population (73). Although disappointing at a first glance, these data are quite relevant. Knowing which SNPs (and genes) have little or no clinical importance on a particular disease helps to refine our choices and redirect new studies into variants and pathways relevant to the field.

MiR-122 is abundantly expressed in hepatic cells (85–87) and markedly influences the clinical course of HCV infection (86, 88). Some attempts to explain this influence have focused on genetic variants that affect miR-122 expression. For instance, Urban et al. (89) evaluated the relationships between the IFNL4/IL28B C/T SNP (rs12979860) and miR-122 expression in liver samples of HCV-infected patients from the United States presenting distinct ancestry (Asian, African American, Caucasian, and Hispanic). They observed a reduced miR-122 expression in samples of patients showing poor response to the treatment. However, this finding was independent of the IFNL4/IL28B rs12979860 genotype. On the other hand, this SNP may also influence the course of HCV infection independently, once carriers of CT or TT genotypes showed higher levels of interferon-stimulated genes compared to those levels linked to CC genotype (89). Evaluating the same SNP in a small sample of HCV-infected patients, Estrabaud et al. (86) observed an increased miR-122 expression in the liver of CC genotype carriers. In this same context, Spaniel et al. (59) reported a reduced miR-122 expression in non-tumor liver samples of HCV-infected individuals, a finding also linked to another SNP of *IFNL4/IL28B* gene: G/T rs8099917. In this study, the TG genotype was associated with a lower expression of miR-122 in non-tumor liver samples of HCV-infected Japanese individuals (59). Moreover, evaluation HCV-infected patients, Su et al. (90) found an association between TT genotype of IFNL4/IL28B rs8099917 and high levels of serum miR-122. In agreement with results from population studies, an *in vitro* assay suggested that both IFNL4/IL28B rs12979860 and IFNL4/IL28B rs8099917 modulate the course of HCV infection, although how exactly this modulation happens is still not understood (87). However, as often is the case, there are conflicting data from studies that do not corroborate these associations (88, 91, 92).

The IFNL3/IL28B A/C SNP (rs4803217) affects the binding of HCV-induced miRNAs (miR-208b and miR-499a-5p) with the *IFNL3* mRNA (93, 94). According to McFarland et al. (93), this phenomenon has important implications for the HCV pathogenesis. Specifically, the G allele of IFNL3/IL28B rs4803217 impairs the activity of both these miRNAs, promoting a high expression of *IFLN3*. As a consequence, the G allele contributes to HCV clearance while the T allele favors (or is neutral) the infection process (93, 94). Based on the study of McFarland et al. (93), Tiang (94) highlighted that miR-208b and miR-499a-5p are potential targets for therapy against HCV infection. Posteriorly, the functional effects of IFNL3/IL28B rs4803217 on miR-208b and miR-499a-5p were challenged by other investigations (95, 96), since it was suggested that the influence of IFNL3/IL28B rs4803217 on HCV infection is promoted by miRNA-independent mechanisms (96). Thus, more studies regarding the role of IFNL3/IL28B rs4803217 polymorphism and miR-208b and miR-499a-5p on HCV infection are welcome.

**Figure 2:**
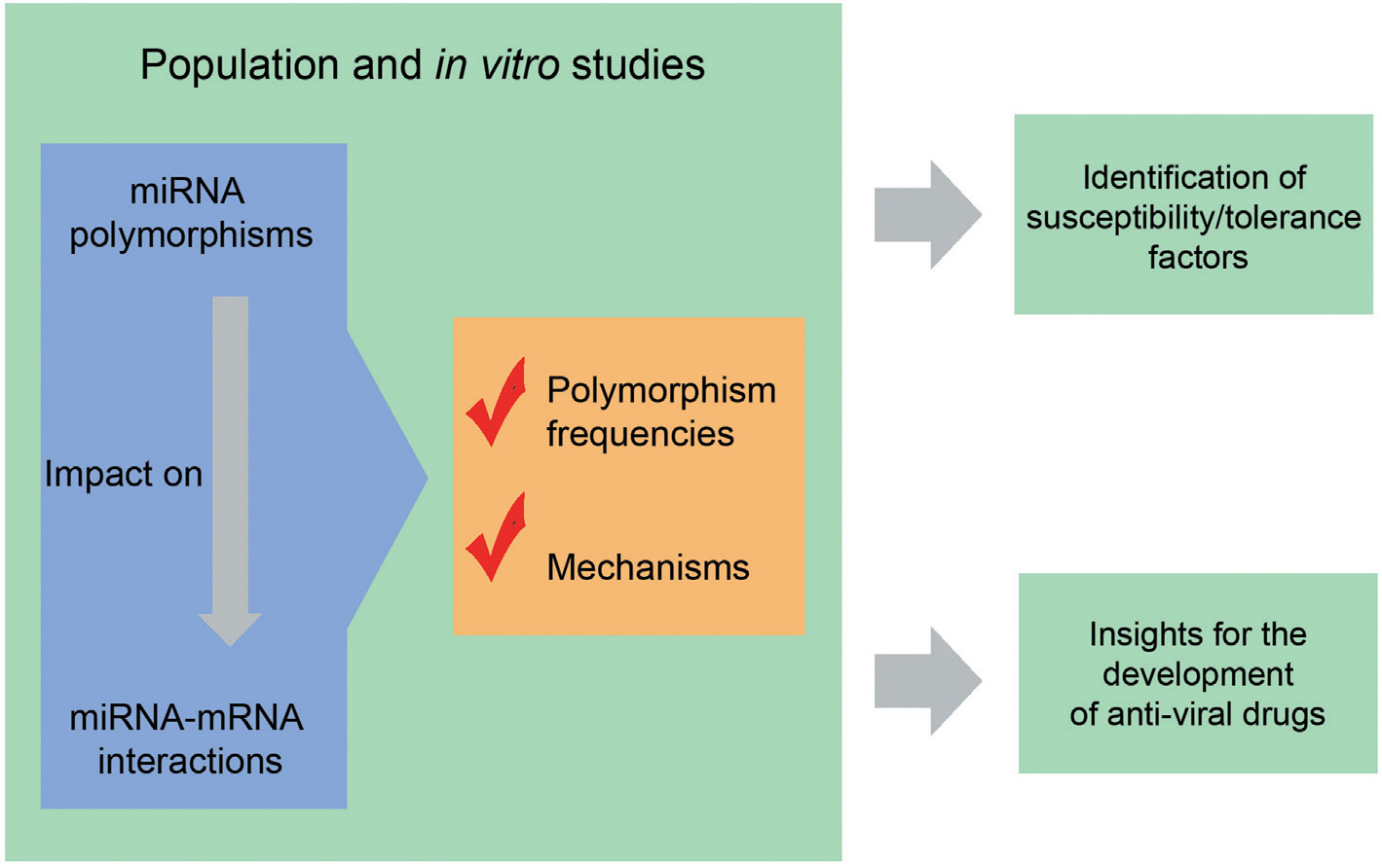
Why to study polymorphisms in the context of viral diseases? Population or *in vitro* studies help to understand which and how polymorphisms impact on microRNA (miRNA)–mRNA interactions. Knowing the population distribution of polymorphisms and relationships of susceptibility/tolerance in the context of viral diseases, make it possible to identify individuals and populations with increased or decreased susceptibility to viral infections, allowing the development of strategies for infection control. In addition, the comprehension of the mechanisms by which miRNA-related polymorphisms influence the outcomes of viral diseases provides insights for the development of new antivirals. See text for references.

Hepatitis C virus uses several strategies to evade the immune system, including miRNAs engagement (84). The disruption of miRNAs that promote HCV infection (for example, those that help HCV to evade the immune system) is a potential therapy for HCV-associated diseases (93). The understanding of how polymorphisms affect this phenomenon can help us in the development of new drugs based on this mechanism (Figure 2). Following these ideas, and based on a study investigating miRNA-101-1 and miRNA-221 expression and their respective SNPs (miR-101-1 C/G/T rs7536540 and miR-221 A/G rs17084733) in an Egyptian population, Shaker et al. (97) proposed the use of miR-101-1 and miR-221 as biomarkers of HCV-associated HCC. However, before applied to the clinical practice, these findings must be validated in different populations in studies with large sample sizes.

Finally, there is some evidence showing that TGFBR1 A/G SNP (rs868) (located at miR-let-7 and miR-98-binding sites) could have an impact on clinical parameters of HCV infection, especially on HCV RNA loads and hepatic inflammation (98). However, in the current scenario, the interaction between this TGFBR1 SNP (rs868) and HCV infection is poorly understood.

### HIV Infection

According to Corbeau (99), different human miRNAs have a close relationship with HIV, both by interacting with HIV RNA as well as with mRNAs of cellular proteins essential for HIV replication. These interactions impact HIV replication, latency, pathogenesis, and also affect the host antiviral immune response. Therefore, the manipulation of these miRNAs expression can be approached as a potential therapeutic tool to mitigate the impact of HIV infection (99). Hariharan et al. (100) suggested that polymorphisms in miRNAs targeting HIV genes may influence the infection progression. However, although the relationship between HIV and miRNAs has already been studied and debated, the effects of miRNA SNPs on HIV-miRNAs interaction have been poorly explored.

One of the few genes evaluated in the context of miRNA and HIV is the *Human Leukocyte Antigen-C* gene (*HLA-C*). The HLA-C ins/del variant (rs67384697) already evaluated in Europeans (101) and in Chinese populations (102), disrupts the binding site of miR-148, impacting the control of HIV infection. The deletion allele was associated to an HIV controller phenotype (low viral loads and high CD4 T^+^ cell counts) and the insertion alleles were associated to an HIV noncontroller phenotype (high viral loads and low CD4 T^+^ cell counts) (101, 102). The HIV controller phenotype was also associated to the CC genotype of HLA-C C/T SNP (rs9264942) (102), although it is worth to note that HLA-C rs67384697 and rs9264942 are in linkage disequilibrium (101, 102). Finally, miR-148a A/G SNP (rs735316) seems to influence the progression of HIV infection by interfering with the expression of HLA-C on the cell surface (103).

Peckham-Gregory et al. (104) evaluated 25 miRNA SNPs in patients with AIDS-associated non-Hodgkin lymphoma (AIDS-NHL) and HIV-infected controls. The authors included in their analyses SNPs located at miRNA coding regions, at miRNA biogenesis genes, and near/within miRNA-binding sites. Among the different results of this study, it worth to highlight: (I) The DDX20 C/T SNP (rs197412) affected miRNA biogenesis and this SNP C allele was associated with an increased risk of AIDS-NHL; (II) The T allele of miR-196a2 C/T SNP (rs11614913) (located at miR-196a2 coding region) was linked to a decreased risk of central nervous system (CNS) AIDS-NHL; (III) The T allele of HIF1A-AS2 C/T SNP (rs2057482) was associated with an increased risk of systemic AIDS-NHL, and (IV) the same allele decreased the risk of CNS AIDS-NHL (104). Of particular interest, HIF1A-AS2 rs2057482 is a variant that creates a binding-site to miR-196a2 (104).

Several CYP2B6 SNPs were evaluated concerning their potential influence in the metabolization of the anti-HIV drug Efavirenz in different contexts (105–109). Among the main findings, the CC genotype of CYP2B6 C/T SNP (rs1042389) was associated to low Efavirenz plasma concentration in Black HIV + individuals from South Africa (110). It is believed that this SNP modifies the expression of *CYP2B6* mRNA since it affects the binding-site of different miRNAs (110, 111). However, the association found in this study was quite weak and the clinical significance of this variant is controversial. Also in South Africa, Maharaj et al. (112) genotyped the miR-27a A/C/G/T SNP (rs895819) in HIV-negative and HIV-positive pregnant women subdivided according to a normotensive or a preeclamptic status. Although the TC/CC genotype of miR-27a rs895819 was associated to increased body mass index (BMI) in the group of HIV-positive women with preeclampsia, it was not associated with preeclampsia susceptibility (112). As miR-27a is an inhibitor of adipogenesis (113, 114) it is believed that miR-27a rs895819 can disrupt this miR-27a action, and then contribute to an increased BMI (112). The association between miR-27a rs895819 and BMI described by Maharaj et al. (112) is quite interesting and deserves to be replicated in other populations with different genetic backgrounds. Posteriorly, the same group described a potential impact of miR-146a C/G SNP (rs2910164) on HIV-positive South African women with preeclampsia (115). Specifically, GC/CC genotypes were associated with a reduced susceptibility to severe preeclampsia in HIV-positive pregnant women on HAART (Highly Active Antiretroviral Therapy). In addition, the miR-146a rs2910164 seems to have an influence on IL-2 levels of pregnant women (115). These results suggest an influence on the progression of HIV-related diseases. However, the patients studied by Maharaj et al. (112, 115) represent a very particular group of women, and before assuming that miR-27a rs895819 or miR-146a rs2910164 have an important influence on the clinical status of HIV-infected individuals from different genetic backgrounds, these SNPs must be studied in distinct populations (infected and non-infected by HIV) in studies recruiting men and women with different health status.

Finally, the A allele of TREX1 A/G SNP (rs3135945), a variant from a gene which encodes a restriction factor against HIV-1, was associated with higher susceptibility to HIV infection in a Caucasian cohort evaluated by Pontillo et al. (116). Since this SNP does not induce aminoacid sequence change, the authors hypothesized that a miRNA-mediated mechanism could explain how TREX1 rs3135945 impacts on HIV infection (116).

### Epstein–Barr Virus

Epstein–Barr virus belongs to the herpesvirus family and is one of the most common viruses in humans, infecting more than 90% of the people worldwide. EBV is well known to cause the infectious mononucleosis (117). However, this virus is also associated with the development of several human tumors (118). EBV infection is a relevant susceptibility factor to nasopharyngeal carcinoma (NPC), and the few data available about the role of polymorphisms in miRNAs and binding target-sites in EBV infection came from studies focused in this type of cancer (32, 119, 120). Actually, the interest in NPC-associated EBV miRNAs emerged from the identification of EBV-encoded viral miRNA in lymphoid malignancies. Given that only a few viral latent proteins are expressed in NPC, researchers have hypothesized that EBV may contribute to cancer development through the viral miRNAs (120). The role of EBV miRNAs is still little known, but studies are pointing to important roles in both viral and cellular gene expression modulation (10, 121, 122).

An interesting case–control association study related to the current topic showed the influence of SNPs within mature-miRNA sequences in NPC susceptibility, assessing a southern China population (32). Further, these preliminary results were validated in a sample from eastern China. Eight SNPs were evaluated in the referred study, including miR-499 rs3746444 C/T, miR-608 rs4919510 C/G, miR-3152 rs13299349 A/G, miR-4293 rs12220909 C/G, miR-4513 rs2168518 C/T, miR-4520a rs8078913 C/T, miR-5579 rs11237828 C/T, and miR-5689 rs9295535 C/T. Among them, only the miR-608 rs4919510 SNP was associated with NPC risk. The presence of the G allele was reported as a susceptibility factor in both Chinese samples, in the two merged populations, and especially in individuals with EBV infection, where the risk effect was more prominent in comparison with individuals not infected. Aiming to evaluate the effects of the miR-608 rs4919510 SNP on NPC tumorigenesis, CNE-1 and CNE-2 cells (both NPC cell lines) were transfected with constructs containing G or C alleles and a soft-agar colony formation assay was performed. In agreement with population-based results, functional analyses indicated that G allele of miR-608 rs4919510 SNP induced more colonies compared to C allele in CNE-2 cells (32). Another investigation also based on population-derived data and functional experiments had previously linked the miR-608 rs4910510 G allele with NPC locoregional recurrence (123). Based on this body of evidence, it is possible to assume that the G allele of miR-608 rs4919510 SNP significantly interacts with EBV, resulting in an increased NPC susceptibility (32). Some of the miR-608 target genes (immune system related genes, or genes associated to DNA repair, metastasis-related, cell death-related, among many others), can have their expression rates altered by the miR-608 rs4919510 SNP (32, 123). Furthermore, EBV can influence host gene transcription (32, 124, 125). In line with this view, Qiu et al. (32) suggested that miR-608 target genes could be directly activated by EBV and the influence of miR-608 rs4919510 SNP on gene transcription could be modified by EBV infection. These complex interaction networks would result in an increased NPC risk, as previously mentioned. Although the impact of miR-608 rs4919510 SNP on host gene expression and the interactions between EVB and host genes are plausible and supported by different data (32, 123–125), it must be characterized in more detail. Finally, the same authors also proposed the use of miR-608 rs4919510 SNP as a marker of NPC risk in a Chinese population (32). Although the above-mentioned data support this suggestion, it is important to replicate these findings in different populations before miR-608 rs4919510 SNP be used as a marker of NPC risk. Further functional studies fully characterizing the effects of this variant on gene regulation are also welcome to help us understand the role of this SNP (32).

Host–EBV interactions have also been investigated revealing that EBV gene regulation can be influenced by host transcriptional regulators. In addition, it was shown that EBV-encoded miRNAs can induce cell transformation in the host (126–128). In fact, EBV-encoded miRNAs have been involved in the regulation of both EBV and human gene expression in NPC. In a study from Lung et al. (120), two nucleotide variations in the primary transcript of miR-BART22 were identified as responsible for its increased biogenesis *in vitro*. This miRNA is coded by EBV and is highly expressed in NPC. Moreover, miR-BART22 modulates the EBV-encoded LMP2A protein expression, which is an oncoprotein recognized by cytotoxic T cells in the host (120). MiR-BART22-induced LMP2A down-modulation may promote EBV-infected cells evasion of the immune system (120). Based on these findings, it is possible to assume that miR-BART22 contributes to EBV pathogenesis. Thus, it makes sense the suggestion that polymorphisms in the miR-BART22 transcript could affect its maturation in NPC, contributing to a higher miR-BART22 expression, which in turn would induce a decreased LMP2A expression facilitating cancer development through the evasion of host immune response (120). Although this is not a case–control association study, it highlights and reinforces the importance of studies about polymorphisms in miRNAs and their binding target-sites in the context of the EBV infection. However, we consider that the most interesting in the study performed by Lung et al. (120) is that it supports the expression control of oncogenic and immunogenic viral proteins by EBV-derived miRNAs. Based on this information, polymorphisms affecting this control potentially play a pivotal role in the NPC development. In this sense, these polymorphisms may be used as models for the study of NPC, once understanding the mechanisms by which polymorphisms in EBV miRNAs interfere with the expression of proteins that modulate tumor biology may provide important insights for the development of NPC therapies. However, to achieve this goal, it is essential to perform functional studies focused on the understanding of the effect of EVB miRNAs and their polymorphisms in pathological and physiological contexts. Since EBV miRNAs modulate the expression of cancer-related proteins (120, 121, 128), they potentially also influence basic cellular physiological mechanisms, such as cell growth, differentiation, and signaling.

A recent characterization of the mRNA and miRNA transcriptome in NPC models (in cell lines that actually harbor EBV), provides a general view about miRNA–mRNA regulation and polymorphisms that can interfere in such regulation (127). This approach represents an interesting starting point for planning new studies about polymorphisms within miRNAs or miRNA target-sites potentially related to EBV infection.

Finally, we call attention to the need for conducting studies involving the characterization of EBV miRNAs, once information on this subject is still scarce. From the characterization of these miRNAs, it will be possible to deepen the investigations of polymorphisms found in sequences of EBV miRNAs. Although NPC is a disease of great relevance, it is also essential do not neglect other EBV-related diseases.

### Human Papillomavirus

Studies encompassing miRNAs and HPV infection are incipient. Actually, a search in article databases returned only 12 articles focusing on genetic polymorphisms related to miRNAs and HPV, being the vast majority on HPV-related cancer development, progression, and prognosis. In this sense, miRNA-related polymorphisms that could modulate immune response and viral restriction, as well as cell cycle, proliferation and death, related to HPV infection often will be evaluated considering tumoral clinical outcomes.

Analyzing an Italian cohort of patients with penile squamous cell carcinomas (PSCCs), Peta et al. (129) investigated the association of a common functional miR-146a C/G SNP (rs2910164) with risk to cancer development. The frequencies of miR-146a rs2910164 genotypes in PSCCs patients, as well as its expression levels, were not different from the distribution observed in the general population, although miR-146a targets various genes that control of immune response, inflammation, cell proliferation, differentiation, and metastasis formation. Considering their potential effects, miRNA expression might act as a two-edged sword, being its upregulation related to immunosuppressive effects and its downregulation associated to cell proliferation and metastases development (129, 130). The authors found an inverse correlation regarding the expression levels of miR-146a and the expression of epidermal growth factor receptor (EGFR), a well-established target of miR-146a. The activation of EGFR pathways is known to increase keratinocytes proliferation and migration, and to be related to HPV-mediated cell immortalization and transformation (131). In fact, miR-146a expression levels were lower in high-risk HPV-positive than in HPV-negative patients, although that difference was not statistically significant. This lower expression was also observed in HPV-positive carcinoma cell lines when compared to cultures from healthy cells. Thus, the authors suggested that HPV-16 E6 downregulates miR-146a expression, leading to an overexpression of EGFR, increasing, this way, the risk of cancer development (129).

Revathidevi et al. (132) studied the effect of a deletion in the *APOBEC3* gene cluster in an attempt to associate HPV infection and cancer development in a South Indian population. This polymorphism corresponds to a deletion of a 29.5-kb fragment, removing sequences from the fifth exon of *APOBEC3A* to the eighth exon of *APOBEC3B*. The polymorphic transcript encompasses the coding sequence of APOBEC3A and the 3′UTR *APOBEC3B*. The APOBEC3A/3B deletion polymorphism has been associated to poor HPV restriction and carcinogenesis promotion (133). Since APOBEC3A/3B deletion involves 3′UTR alterations, Revathidevi et al. (132) hypothesized that miRNA-mediated posttranscriptional regulation could be important to the *APOBEC3A/3B* overexpression. Nevertheless, no association between APOBEC3A/3B deletion polymorphism and the cancer development was observed, independently of the cancer type (they evaluated breast, cervical, and oral cancer samples), contrasting with previous studies that found associations of this polymorphism with cancer development (134, 135). Interestingly, the expression of an *APOBEC3B* miRNA that could regulate *APOBEC3A/3B* fusion transcript (miR-34b-3p) was downregulated in cervical tumor samples, suggesting that this miRNA may lead to a loss of miRNA repression and a consequently increased expression level of the APOBEC3A/3B protein.

In a Chinese population, Wu and Zhang (136) studied the association of miR-124 C/G (rs531564) with susceptibility to HPV infection and cervical cancer. The authors found that the miR-124 rs531564 G allele and the CG genotype were associated to a reduced risk of HPV infection, compared to the C allele and the CC genotype. Additionally, the miR-124 rs531564 G allele was described as associated to a reduced susceptibility to cervical cancer, corroborating other studies (137, 138). The authors hypothesize that the miR-124 rs531564 G allele promotes the expression of a mature form of this miRNA, leading to a lower risk of HPV infection and a subsequent reduced risk to cervical cancer.

Zhou et al. (139), studying another Chinese population, described the influence of miR-218 on HPV-related cervical cancer. Two polymorphisms were investigated: one at the primary-miR-218 (pri-miR-218) A/G SNP (rs11134527), and the second located at the 3′UTR of *LAMB3* (laminin-5 β3) gene (rs2566, C/T), a known target of miR-218, which is suppressed by the HPV-16 E6 protein. In fact, the expression of LAMB3 is augmented in the presence of the HPV-16 E6 oncoprotein and this effect is regulated through miR-218 (140). Laminin-5 plays an important role on the development of cervical lesions, and has been indicated as a marker of invasiveness (141). The authors evidenced an association of the pri-miR-218 rs11134527 variant homozygote GG genotype with a decreased susceptibility of cervical cancer development, as compared to the AA genotype. Regarding the LAMB3 rs2566 polymorphism, Zhou et al. (139) showed that the presence of the T allele, in a dominant model, was significantly associated to a higher risk of cervical cancer. Moreover, when these susceptibility variants were present together, the risk of cervical cancer was significantly higher, in a dose-dependent manner (139).

Several authors have studied the effect of miRNA-related polymorphisms on the development of oral squamous cell carcinoma (OSCC) and its variations [oropharynx (SCCOP) and oral cavity (SCCOC)] (142–147). These cancer types respond by the majority of head and neck malignant tumors worldwide and are highly associated to HPV infection. Song et al. (143) described the effect of four miRNA SNPs [miR-146 G/C (rs2910164), miR-149 C/T (rs2292832), miR-196 C/T (rs11614913), and miR-499 C/T (rs3746444)] in HPV-16 seropositivity and OSCC in a population from the United States. No statistically significant associations of these polymorphisms were observed. In fact, also an absence of association was observed between miR-146 rs2910164 and miR-196 rs11614913 with OSCC overall survival rates in a European cohort (144). However, Song et al. (143) described that, according to HPV-16 seropositivity, miRNA SNPs profiles could play a role in OSCC. Compared with individuals both miR-146 rs2910164 GG genotype and HPV-16 negative, those both GG genotype and HPV-16 positive presented an augmented risk of OSCC, and the susceptibility was even higher when the C allele was present. Similar results were obtained for the associations between miR-149 rs2292832 (CC genotype), miR-196 rs11614913 (C allele presence), and miR-499 rs3746444 (C allele presence) SNPs and risk of HPV-16-associated OSCC (143). Specifically to SCCOP, Guan et al. (142) described in the same population that compared to miR-146 rs2910164 CG/CC and miR-196 rs11614913 CC genotypes, individuals carrying both miR-146 rs2910164 GG and miR-196 rs11614913 CT/TT genotypes were significantly associated to a better overall, disease-specific, and disease-free survival in HPV-positive tumors (142). In line with these data, Song et al. (143) found that individuals with the combined miR-146 rs2910164 CG and CC genotypes had a higher risk of SCCOP than individuals with the GG genotype, and individuals with the miR-499 rs3746444 combined CT and CC genotypes had a higher risk of SCCOP than individuals with the TT genotype (143).

The same research group also studied the effect of polymorphisms located in putative miRNA-binding sites in the 3′UTR of genes related to DNA repair pathways in SCCOP recurrence in HPV-16-positive tumors (147). The authors found that only BRCA1 C/T (rs12516) and RAD51 A/G (rs7180135) SNPs were associated with SCCOP incidence. Patients with the variant genotypes of BRCA1 rs12516 (CT/TT) and RAD51 rs7180135 (AG/GG) SNPs presented a significantly lower susceptibility of disease recurrence as compared to patients with the corresponding common homozygous genotypes. Moreover, BRCA1 rs12516 CC genotype had a significantly higher BRCA1 protein expression, compared to CT/TT variant genotypes and RAD51 rs7180135 AA genotype had a borderline significant association to a higher expression of RAD51 protein, compared to RAD51 rs7180135 AG/GG variant genotypes. BRCA1 rs12516 SNP has been described as a potential binding site of several miRNAs, two of them (miR-118 and miR-639) have already been associated to cancer risk, while RAD51 rs7180135 SNP was described as a potential binding site of miR-197. Other miRNAs were also described to target *RAD51* gene (miRNA-182, miR-155, miR-103, and miR-107), showing the importance of further studies to determine how this gene expression could be regulated.

Yuan et al. (146) and Zhang et al. (145) described in the same population that ins/del polymorphisms in 3′UTR of *E2F1* and *IL-1α* genes, respectively, are associated to OSCC HPV related (145, 146). Both genes have been described as important on the control of cell death and proliferation and variations on 3′UTR are supposed to impact on miRNA targeting. The authors showed that the E2F1 rs3213180 ins/del and ins/ins and the IL-1α 3′UTR (rs3783553) del/del genotypes, jointly to HPV seropositivity, are associated to a higher susceptibility to HPV-related OSCC. The E2F1 rs3213180 is the only miRNA-binding site described at E2F1 3′UTR that may affect E2F1 expression levels (148), while IL-1α rs3783553 interfere on miR-122-binding site, regulating IL-1α expression levels (149). In fact, Zhang et al. (145) described a significantly increased expression of IL-1α in patients with del/del genotype as compared to ins/ins and ins/del genotypes (145).

Another crucial regulatory gene is the *Cyclin-dependent kinase 6* (*CDK6*), which is associated with cell cycle and tumorigenesis. Various miRNAs are reported to be involved in CDK6-mediated tumorigenesis, such as miR-145, miR-320, and miR-29. In a Chinese population, Ye et al. (150) studied for the first time the effect of five genetic variations in the 3′UTR of *CDK6* gene (rs8179 G/A, rs4272 A/G, rs42033 A/T, rs42035 T/C, and rs42377 G/A) on susceptibility to precancerous cervical lesions. The authors found that the rs8179 A and rs42033 T alleles were associated to a lower risk to develop precancerous cervical lesions and had an antagonistic interaction with the HPV infection. This lower susceptibility to cervical lesions was also observed to rs8179 GA, compared to AA genotype and rs42033 AT, compared to AA genotype, after adjustments for HPV infection and others clinical and demographic characteristics. Strong linkage disequilibrium values were observed between rs8179, rs4272, rs42033 and rs42377 and the haplotype AGTA was significantly associated to a reduced risk to precancerous cervical lesions when compared to GAAG haplotype (150).

To our knowledge, only one study described polymorphisms in HPV related to miRNA-binding sites. Mandal et al. (151) showed that polymorphisms located at a short non-coding region (NCR2), commonly present between HPV E5 and L2 open reading frames, could lead to a loss of human miRNA sites. Through *in silico* analysis, the authors identified binding sites at the NCR2 region in HPV-16 corresponding to 14 human miRNAs (miR-3148, miR-3174, miR-3613-3p, miR-3916, miR-495, miR-548a-5p, miR-548b-5p, miR-548c-5p, miR-548d-5p, miR-548h-5p, miR-548i-5p, miR-548j-5p, miR-548w-5p, and miR-548y-5p). Moreover, the authors revealed the occurrence of an SNP (T4228C) in the NCR2 of a variant isolate, which could lead to loss of 9 miRNA-binding sites in the corresponding transcripts (151).

## Emerging Topics

**Figure 3:**
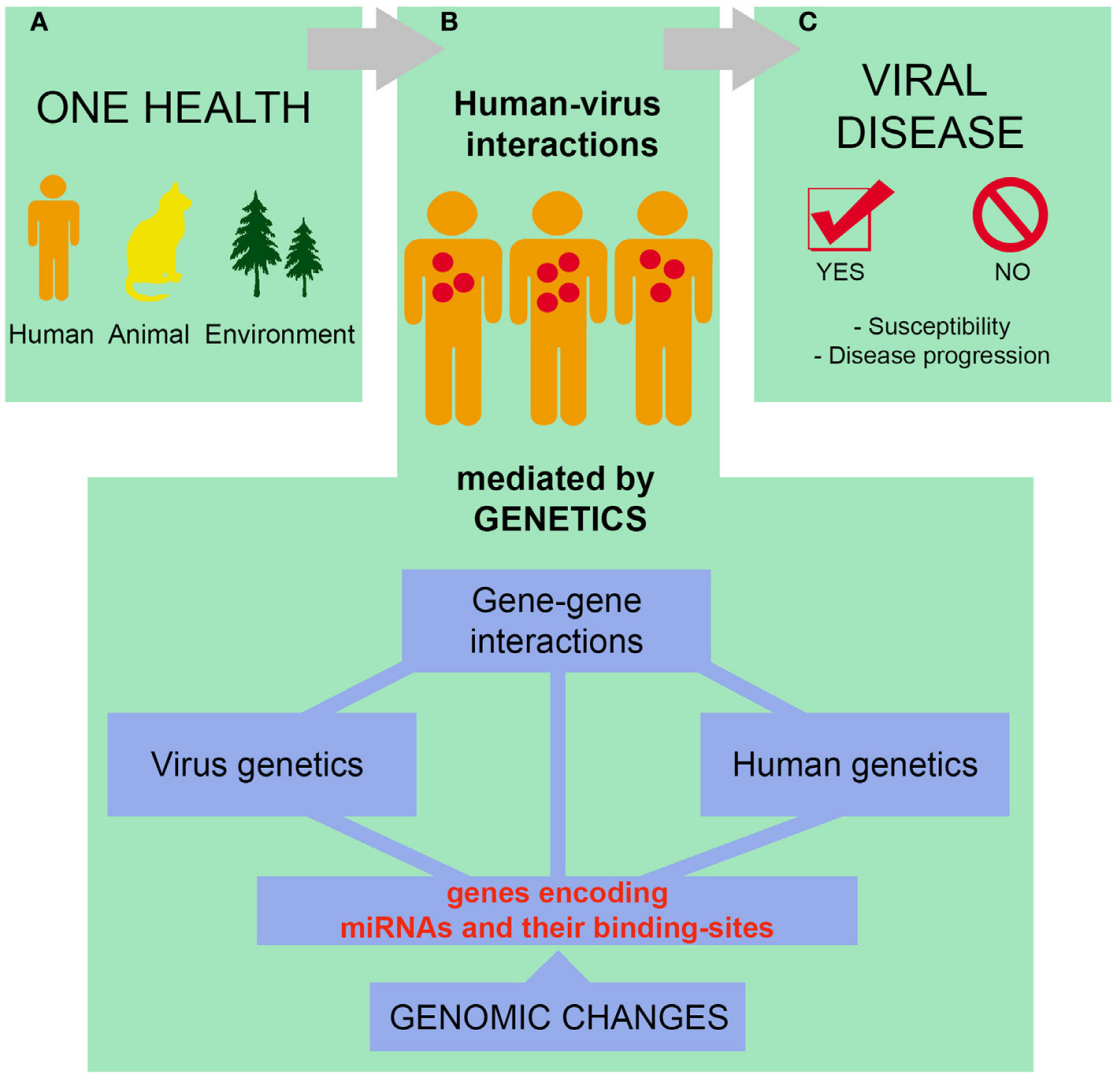
Susceptibility to infections and progression of viral diseases are complex processes that must be thought within the context of One Health. Human, non-human, and environmental factors define whether a given individual will come into contact with a particular virus and the consequences of such interaction **(A)**. Of note, human–virus interactions are mediated by both host and viral genetic factors, including microRNAs (miRNA) and miRNA-related polymorphisms **(B)**. The complex interactions mentioned in **(A,B)** influence the susceptibility to infections as well as the progression of viral diseases **(C)**. See text for references.

Currently, the prevention and control of infectious diseases should be approached as a global strategy, in which human, animal, and environmental factors are integrated (152–154), allied to the evaluation of the genomic characteristics of the pathogens (155, 156). Among the human factors that should be taken into consideration, the investigation of genetic features that alter the susceptibility to infection and progression of viral diseases is essential for the prevention and clinical management of infectious diseases in specific human populations (157–159) (Figure 3).

SNPs are the most common form of genetic variation in the human genome (160). For many years human SNPs have been highly studied worldwide. However, it is necessary to focus those studies also on viral genetic variants and try to understand how these variants affect the action of miRNAs, for example. For instance, some evidence indicates that SNPs have a relevant impact on the biogenesis and action of Kaposi’s sarcoma-associated herpesvirus (KSHV) miRNAs (161). KSHV infection is highly linked to Kaposi’s sarcoma development (162). According to a set of *in vitro* analyzes based on clinical observations, Han et al. (161) have shown that different SNPs in KSHV miRNAs alter the expression level of these miRNAs, as well as modify their processing and silencing activities. These changes may alter KSHV pathogenesis, potentially impacting Kaposi’s sarcoma development (161). Of note, a number of polymorphisms (SNPs, deletions, and insertions) in KSHV miRNAs have already been observed in pri-miRNAs, pre-miRNAs, and mature miRNAs (163–165). Looking at KSHV-related diseases, these polymorphisms affect miRNAs maturation and some of them may also affect the risk of Kaposi’s sarcoma development in patients with AIDS (164), although as a whole the effects of these polymorphisms on KSHV pathogenesis are poorly understood. Furthermore, it has been shown that polymorphisms in KSHV miRNAs influence the risk for the development and the pathogenesis of multicentric Castleman disease and KSHV-associated inflammatory cytokine syndrome, diseases also linked to KSHV infection (165).

The number of KSHV miRNAs described in the literature is increasing (166–168). Similarly, the effects of them on immune response, KSHV pathogenesis and Kaposi’s sarcoma development have already been described (166–172). The implications of KSHV miRNA SNPs on the miRNA processing are also being characterized (164, 173). However, there is much to be explored about the SNPs located in KSHV miRNAs. Once these SNPs are well characterized, we will better understand their effects on Kaposi’s sarcoma development and other KSHV-related diseases.

Another interesting example of viral miRNA variant involves the human T cell leukemia virus-type 1 (HTLV-1); Host miR-28-3p is an inhibitor of HTLV-1 replication and infection (31). The Thr-to-Cys (AAT-to-AAC) polymorphism in ATK-1 HTLV strain (subtype 1A) disrupts the miR-28-3p target site. This disruption affects the anti-HTLV action of miRNA-28-3p. However, miR-28-3p target site is highly conserved in the HTLV-1 subtypes B and C, and therefore, this miRNA has a therapeutic potential in strategies to control HTLV-1 infection (31).

The role of miRNA SNPs in viral infections other than those previously approached in the present review has been poorly explored, although some studies can be cited. For example, Misra et al. (174) evaluated the influence of the following host miRNAs SNPs on human cytomegalovirus (HCMV) infection: miR-146a C/G (rs2910164), miR-196a2 C/T (rs11614913), miR-499a C/T (rs3746444), and miR-149 C/T (rs2292832). In brief, with exception of miR-149 rs2292832, mutant genotypes of the other three SNPs were linked to increased risk of symptomatic HCMV infection. Multifactor Dimensionality Reduction analysis (applied to access SNP–SNP interactions) indicated an association between increased risk of symptomatic HCMV infection with the four interaction models tested (174). This result indicates that miRNA SNPs play a relevant role in the pathogenesis of HCMV. However, to the best of our knowledge, no other study focusing on the role of miRNA SNPs in HCMV infection was performed, making this a blank spot to further studies.

Finally, we should highlight the triad (I) exosomes, (II) miRNAs, and (III) viral infections. Exosomes are extracellular nanovesicles originated from multivesicular bodies. These vesicles have drawn attention from the scientific community due to their ability to transport protein, lipid, and genetic components between different cells in a highly regulated manner (175). Moreover, a large body of evidence showed that host and viral miRNAs are one of the major types of components transported by exosomes (176, 177). Exosomes have a relevant immunomodulatory action (176, 178) and have been shown to strongly interact with different viruses, such as HIV (179, 180), Ebola (181), HBV (182), and others (183). Taking into account the therapeutic potentials of the exosomes-mediated miRNA delivery pathway supported by recent findings (184, 185), in the near future, it will be possible to modulate the exosomes-mediated miRNA trafficking aiming to mitigate viral infections. In addition, a therapeutic control of exosomes-mediated miRNA delivery could be used to induce, or avoid, similar effects to those triggered by miRNA SNPs. However, in order to these therapeutics become a reality, one must to disclosure the influences of the miRNAs transported by the exosomes in viral diseases, and to decipher how miRNA SNPs modulate the pathogenesis of different viruses.

## Conclusion and Perspectives

**Figure 4:**
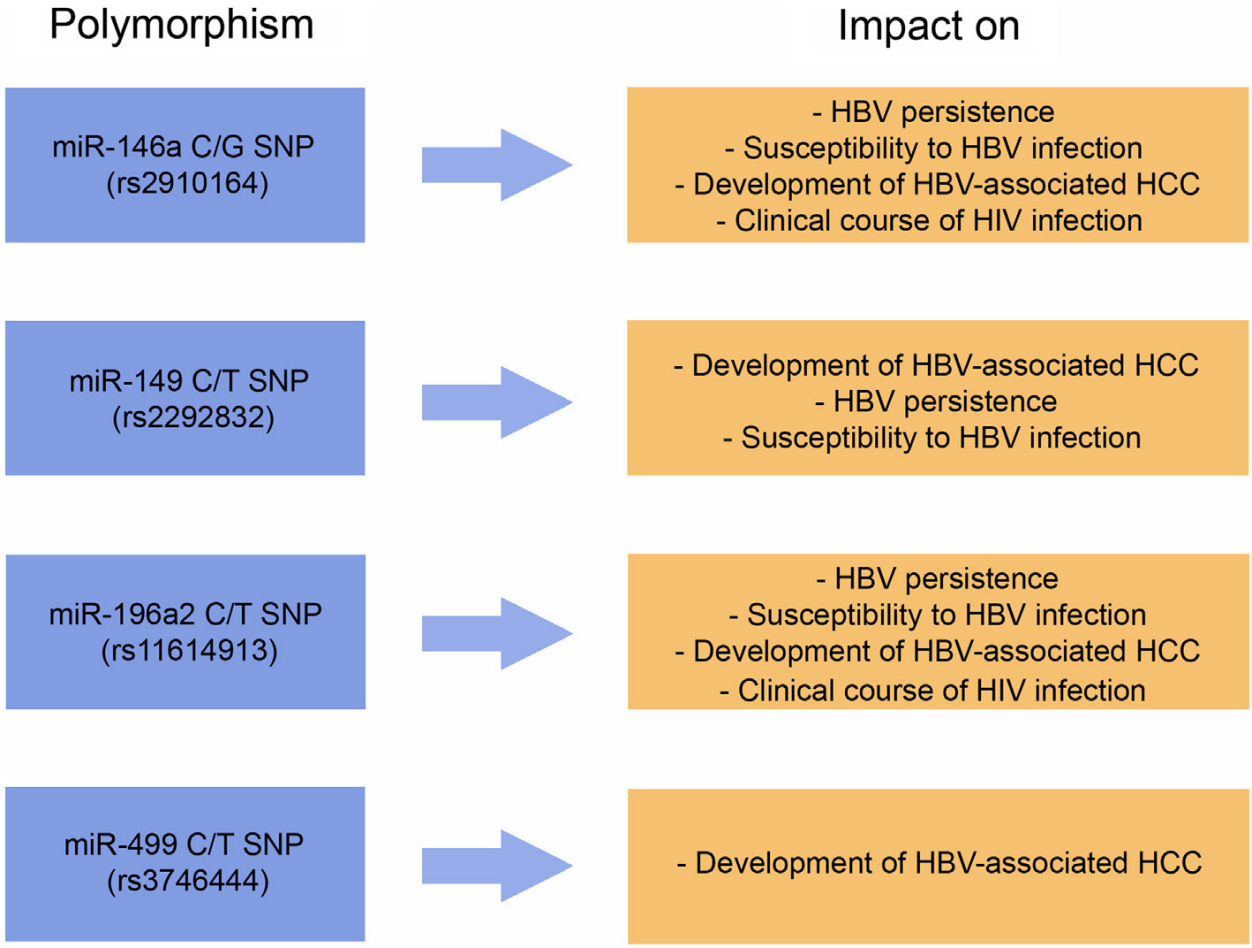
Summary of the main miRNA-related polymorphisms discussed in this review and their impacts on viral diseases. See text and Tables for references.

Based on the articles discussed in this review, we present in Figure 4 the miRNA SNPs that were studied with greater robustness in the context of the viral infections, as well as the influences of these selected SNPs on viral diseases. In summary, development of HBV-associated HCC is influenced by the following polymorphisms: miR-146a G/C SNP (rs2910164), miR-149 C/T SNP (rs2292832), miR-196a2 C/T SNP (rs11614913), and miR-499 C/T SNP (rs3746444). In addition, in Table 2 the main findings of the studies addressing HCV, HIV, EBV, and HPV infections are presented. The study of genetic variants located in miRNA genes or in genes of miRNA-binding sites is incipient. As research continues, new SNPs will be described and new influences of miRNA SNPs on viral diseases will be reported. It is also possible that future investigations will redirect the discussions about the biological or clinical significance of the variants presented in this review. On the other hand, part of these results can be strengthened with the completion of new studies. Of note, the majority of the studies mentioned in this review are punctual and specific of particular populations. Studies investigating miRNA SNPs in Asian populations highly outnumber the studies performed with non-Asian populations. Thus, it is essential to investigate the frequencies of miRNA SNPs in worldwide populations in order to gather better data about susceptibility and progression of viral diseases in different ethnic/genetic backgrounds. The identification of SNPs that influence characteristics of susceptibility or clinical outcome in different populations will be essential for the understanding of the biological significance of such genetic factors. Thus, new meta-analyses will be essential to establish with robustness the effects of those SNPs on infectious diseases. We also draw attention to the fact that most of the studies within the scope of this review investigated human miRNA SNPs. It is necessary to explore the importance of viral miRNAs and their variants on the clinical course of infectious diseases. Also of great importance, the prevalence of viruses of different genotypes is variable around the world, which may or may not complicate what this review is dealing with. Thus, the evaluation of viral genetic characteristics is significant in population-based studies focused on miRNA SNPs. In this sense, when virus genotype is available, this information must be considered during the interpretation of the studies here mentioned.

**Table 2:** Main microRNAs (miRNA)-related polymorphisms showing statistically significant influence on Epstein–Barr virus (EBV), hepatitis C virus (HCV), human immunodeficiency virus (HIV), and Human Papillomavirus (HPV) infections.

Virus	miRNA or miRNA-binding site^a^	Polymorphism^b^	Influence on	Population	Reference
EBV	miR-608	C/G rs4919510	EBV-related nasopharyngeal carcinoma (NPC); NPC risk	Chinese	Qiu et al. (32)

HCV	miR-208b- and miR-499a-5p-binding sites	A/C rs4803217	HCV clearance	*In vitro* experiment	McFarland et al. (93)
	miR-let-7- and miR-98-binding sites	A/G rs868	HCV loads; hepatic inflammation	Polish	Sajjad et al. (98)

HIV	miR-148-binding site	ins/del rs67384697	HIV loads	European descendant	Kulkarni et al. (101)
			HIV loads; CD4 T^+^ cell counts	Chinese	Blais et al. (102)
	miR-148-binding site	C/T rs9264942	HIV loads; CD4 T^+^ cell counts	Chinese	Blais et al. (102)
	miR-148a	A/G rs735316	Progression of HIV infection	European descendant	Kulkarni et al. (103)
	miRNA biogenesis	C/T rs197412	AIDS-associated non-Hodgkin lymphoma risk	American	Peckham-Gregory et al. (104)
	miR-196a2	C/T rs11614913	Central nervous system (CNS) AIDS-associated non-Hodgkin lymphoma risk	American	Peckham-Gregory et al. (104)
	miR-196a2-binding site	C/T rs2057482	CNS and systemic AIDS-associated non-Hodgkin lymphoma risk	American	Peckham-Gregory et al. (104)
	miR-27a	A/C/G/T rs895819	HIV/AIDS-associated nutritional status	African descendant	Maharaj et al. (112)
	miR-146a	C/G rs2910164	HIV-related diseases (particularly preeclampsia)	African descendant	Maharaj et al. (115)

HPV	miR-146a	C/G rs2910164	HPV-related cancer	Chinese	Guan et al. (142); Song et al. (143)
	miR-149	C/T rs2292832	HPV-related cancer	Chinese	Song et al. (143)
	miR-196a	C/T rs11614913	HPV-related cancer	Chinese	Guan et al. (142); Song et al. (143)
	miR-499	C/T rs3746444	HPV-related cancer	Chinese	Song et al. (143)
	miRNA-binding sites	C/T rs12516 and A/G rs7180135	HPV-related cancer	Chinese	Zhu et al. (147)
	miRNA-binding sites	ins/del rs3213180	HPV-related cancer	Chinese	Yuan et al. (146)
	miR-122-binding site	ins/del rs3783553	HPV-related cancer	Chinese	Zhang et al. (145)
	miR-218	A/G rs11134527	HPV-related cancer	Chinese	Zhou et al. (139)
	miR-218-binding site	C/T rs2566	HPV-related cancer	Chinese	Zhou et al. (139)
	miRNA-binding sites	A/G rs8179 and A/T rs42033	HPV-related cancer	Chinese	Ye et al. (150)
	miR-124	C/G rs531564	HPV infection and cervical cancer	Chinese	Wu and Zhang (136)

*^a^Seed or regulatory region*.

*^b^Polymorphism quotations were standardized according to the Single Nucleotide Polymorphism Database (dbSNP) of NCBI (https://www.ncbi.nlm.nih.gov/snp/), based on the reference SNP cluster (rs#) of each polymorphism*.

Finally, directing further investigations to the SNPs discussed here may provide important insights for the development of new therapies against infectious diseases based on inhibitors or stimulators of the action of miRNAs. As discussed earlier, knowing how SNPs alter biogenesis, processing or the action of miRNAs may also be useful for the development of antiviral therapies or for the treatment of complications caused by viral infections. Technologies focused on the delivery of miRNAs in an accurate manner, as engineered exosomes, will also contribute to the success of these therapies. We believe that we are close to experiencing a boom of the miRNA-based therapies.

## Author Contributions

JE, FZ, and RG reviewed the studies and wrote the manuscript. JC wrote and reviewed the manuscript.

## Conflict of Interest Statement

The authors declare that the research was conducted in the absence of any commercial or financial relationships that could be construed as a potential conflict of interest.
